# Understanding middle‐aged and older adults' first associations with the word “cancer”: A mixed methods study in England

**DOI:** 10.1002/pon.4569

**Published:** 2017-11-07

**Authors:** Edelyn Agustina, Rachael H. Dodd, Jo Waller, Charlotte Vrinten

**Affiliations:** ^1^ Department of Behavioural Science and Health UCL London UK

**Keywords:** attitude, cancer, cross‐sectional studies, fatalism, fear, mixed methods, oncology, qualitative research, worry

## Abstract

**Objective:**

Cancer is still widely feared and often associated with death. Fatalistic beliefs adversely affect help‐seeking for cancer symptoms and engagement in cancer prevention. This study aims to understand middle‐aged and older adults' first association with the word “cancer” and their relationship with sociodemographic factors, cancer fear, and cancer information avoidance.

**Methods:**

We conducted a cross‐sectional survey of 1464 community‐based adults aged 50 to 70 living in England in April 2015. First associations with cancer were measured qualitatively and analysed using content analysis. We used binary logistic regression to analyse associations between the most common first association of cancer and sociodemographic characteristics, cancer fear, and cancer information avoidance.

**Results:**

Cancer was most commonly associated with “death” (26%). Respondents with lower levels of education, living in the Midlands or North of England where cancer mortality is higher, or with close friends or family members with a cancer history, were more likely to associate cancer with death. Cancer fear was significantly associated with death associations, but cancer information avoidance was not.

**Conclusions:**

Despite improved cancer outcomes, middle‐aged and older adults often associate cancer with death. Further efforts to decrease fatalistic associations in this age group may be needed.

**Abbreviations**
ABACUS: Attitudes Behaviour and Cancer UK Survey
BAME: Black, Asian, and Minority Ethnic
CSM: Common‐sense model of illness representations
GCSE: General Certificate of Secondary Education
OR: Odds ratio

## 1 BACKGROUND

Cancer survival has improved for most cancers in many high‐income countries,^1^, ^2^ but incidence continues to rise, particularly in older and more deprived groups, and public attitudes about cancer remain negative. In the UK, nearly a quarter of adults aged 50+ believe “a diagnosis of cancer is a death sentence,”^3^ while cancer makes nearly two‐thirds of US adults automatically think of death.^4^, ^5^ About a quarter feel that prevention of cancer is not possible,^5^ and one in two UK adults believe cancer treatment is worse than cancer itself.^3^ These studies show that negative beliefs about cancer are still prevalent in the general population.

Negative beliefs about cancer adversely affect cancer‐related behaviours. Cancer fatalism (the belief that cancer is inevitably fatal^6^), has consistently been shown to deter individuals from attending cancer screening,^7^, ^8^ help‐seeking for possible cancer symptoms,^9^ and attending to cancer‐related information.^10^, ^11^, ^12^ Such fatalistic beliefs are more prevalent in certain subgroups of the population, including those from more deprived backgrounds.^3^ In addition, although older age is a major uncontrollable risk factor for cancer, it is estimated that 43% of cancers could be prevented through healthier lifestyle choices,^13^ and avoidance of cancer information due to fatalistic beliefs may thus represent a missed opportunity for cancer prevention. It is therefore important to monitor negative beliefs about cancer in the population and identify subgroups that are more likely to hold these beliefs and may be deterred from cancer prevention and early diagnosis.

However, many of the studies that have quantitatively monitored these negative beliefs have also measured *positive* beliefs about cancer, and endorsement rates of these beliefs tend to be high. For example, 80% to 90% of US and UK adults believe that cancer can often be cured, and there is near‐universal agreement that presenting promptly with symptoms, or getting checked regularly for cancer, could improve survival chances.^3^, ^4^, ^5^ This suggests that a substantial proportion of the general population may concurrently hold positive and negative beliefs about cancer.

A disadvantage of these quantitative population‐based studies is that they use prompted methodology to measure agreement with predefined statements. Previous research suggests using prompted (recognition) versus unprompted (recall) measures of cancer‐related knowledge yields very different results,^14^ which may also be the case with attitudes. In addition, such quantitative studies cannot determine whether it is the positive or the negative beliefs about cancer that come to mind more readily. A qualitative study of 30 UK adults provides a more in‐depth analysis of the balance of negative and positive beliefs.^15^ The study found the majority of interviewees had initial reactions to cancer that were fearful and fatalistic, although these “gut responses” were often followed by more optimistic and hopeful ones. These unprompted responses to cancer may affect our basic tendency to approach or avoid. For example, associations of cancer with death may elicit fear and avoidant behaviours, while associations of survival and behavioural control may elicit an openness to learn more about cancer prevention and early detection. The content of unprompted beliefs about cancer may thus be important to inform cancer control messages and strategies.

The aim of the current study was to extend previous qualitative findings,^15^ using a mixed‐methods design to examine people's first association with the word “cancer” in a large, population‐based study of middle‐aged and older adults, for whom the threat of cancer is relevant due to their age. We also examined the sociodemographic distribution of the most common first association (which we found to be “death”), and its relationship with cancer fear and cancer information avoidance. On the basis of previous studies,^6^, ^9^, ^16^, ^17^, ^18^, ^19^ we hypothesised that those from lower socioeconomic status and ethnic minority backgrounds would be more likely to have fatalistic cancer beliefs. We predicted more negative beliefs in the Midlands and the North of England, where cancer mortality is higher than in the South.^20^ Expectations of cancer outcomes may also vary by experience of cancer in others, although previous findings in relation to this have been mixed.^3^, ^15^, ^16^, ^18^, ^21^

## 2 METHODS

### 2.1 Design

Data were obtained from the second Attitudes, Behaviour, and Cancer‐UK Survey (ABACUS), a series of 4 population‐based surveys designed to assess attitudes to cancer and cancer screening in England in 2014 to 2016.^12^ The fieldwork was conducted by TNS Research International using home‐based computer‐assisted personal interviewing in April 2015. Residences were selected based on random location sampling using the 2011 census small‐area statistics and postcode address file (stratified by Government Office Region and social grade), with quotas set for age, gender, children in the home, and working status at each location.

### 2.2 Participants

Overall, 1464 participants aged 50 to 70 completed the interview. Participants with missing sociodemographic information were excluded from the present analyses, as well as those with a history of cancer, because they were not asked questions about cancer fear to avoid distress. This study was exempt from ethics approval under the UCL Research Ethics Committee guidelines, because it involved “the use of non‐sensitive, completely anonymous […] survey and interview procedures” and “the participants are not defined as ‘vulnerable’ and participation will not induce undue psychological stress or anxiety” (https://ethics.grad.ucl.ac.uk/exemptions.php). All participants provided informed consent.

### 2.3 Materials and procedure

#### 2.3.1 First association with cancer

Respondents' first associations with cancer were assessed qualitatively by asking: “What is the first thing that comes to mind when you think about cancer?” (adapted from^22^). No time or word limit was set for responses. The interviewer recorded the verbatim response in a free‐text box using a stylus pen.

#### 2.3.2 Sociodemographic factors

All other responses were measured quantitatively using simple questions. Sociodemographic variables included age, gender, marital status, ethnicity, education level, and region of residence. Marital status was recorded as married/living as married, single, or separated/widowed/divorced. Due to small numbers in each of the ethnic minority groups, the ethnicity variable was dichotomised as “White” vs “Black, Asian, and Minority Ethnic (BAME)” (including mixed ethnic background). Education level was collapsed into 5 categories (see Table 1). Experience of cancer in close family or friends was assessed by asking, “Have any friends or family members that are close to you ever been diagnosed with cancer?” and scored as “yes” or “no.” Don't know and refused responses were coded as missing throughout.

**Table 1:** Sample characteristics and unadjusted and adjusted odds ratios (OR) of having “death” as first association of cancer versus not by sociodemographic characteristics, cancer fear, and cancer information avoidance (n = 1105)

Characteristic	Sample n (%)	“Death” Associations n (%)	Unadjusted OR (95% CI)	Significance (*P* value)	Adjusted OR (95% CI)	Significance (*P* value)
Age (years) (Mean [SD])	59.92 (6.25)		0.99 (0.97‐1.01)	.241	0.98 (0.95‐1.00)	.061
Gender						
Male	558 (50.5)	167 (29.9)	1.00		1.00	
Female	547 (49.5)	164 (30.0)	0.93 (0.71‐1.22)	.620	0.76 (0.56‐1.04)	.084
Marital status						
Married/living as married	681 (61.6)	189 (27.8)	1.00		1.00	
Single	158 (14.3)	54 (34.2)	1.30 (0.88‐1.91)	.190	1.20 (0.78‐1.87)	.418
Separated/widowed/divorced	266 (24.1)	88 (33.1)	1.33 (0.97‐1.83)	.082	1.33 (0.93‐1.90)	.120
Ethnicity						
White	1033 (93.5)	304 (29.4)	1.00		1.00	
BAME	72 (6.5)	27 (37.5)	1.22 (0.72‐2.06)	.464	1.37 (0.72‐2.63)	.343
Education level						
Bachelor's degree or higher	236 (21.4)	45 (19.1)	1.00		1.00	
A levels/ONC/BTEC or equivalent^a^	236 (21.4)	61 (25.8)	1.48 (0.93‐2.37)	.099	1.39 (0.84‐2.31)	.199
O levels/GCSE or equivalent^b^	281 (25.4)	81 (28.8)	1.85 (1.19‐2.88)	.006	1.93 (1.20‐3.11)	.007
No formal qualifications	288 (26.1)	108 (37.5)	2.73 (1.78‐4.19)	<.001	2.90 (1.80‐4.68)	<.001
Other (includes still studying)	64 (5.8)	27 (42.2)	3.45 (1.87‐6.36)	<.001	2.70 (1.37‐5.30)	.004
Region of residence						
East of England	144 (13.0)	31 (21.5)	1.00		1.00	
North of England	325 (29.4)	111 (34.2)	2.15 (1.31‐3.51)	.002	1.93 (1.15‐3.23)	.013
Midlands	213 (19.3)	65 (30.5)	1.74 (1.03‐2.95)	.004	1.76 (1.00‐3.06)	.047
London	88 (8.0)	33 (37.5)	1.89 (1.00‐3.56)	.049	1.40 (0.65‐3.00)	.386
South of England	335 (30.3)	91 (27.2)	1.35 (0.82‐2.23)	.242	1.26 (0.74‐2.15)	.387
Family/friends diagnosed with cancer						
Yes	799 (72.3)	259 (32.4)	1.00		1.00	
No	306 (27.7)	71 (23.2)	0.69 (0.50‐0.94)	.020	0.67 (0.47‐0.96)	.027
Cancer fear						
No cancer fear	393 (35.6)	89 (22.6)	1.00		1.00	
Moderate cancer fear	512 (46.3)	120 (23.4)	1.02 (0.75‐1.40)	.887	1.13 (0.80‐1.58)	.494
High cancer fear	173 (15.7)	71 (41.0)	2.16 (1.47‐3.18)	<.001	2.21 (1.42‐3.43)	<.001
Don't know/refused	27 (2.4)					
Cancer avoidance						
No avoidance	755 (68.3)	214 (28.3)	1.00		1.00	
Avoids at least one information source	256 (23.2)	88 (34.4)	1.24 (0.90‐1.70)	.192	0.95 (0.67‐1.35)	.772
Don't know/refused	94 (8.5)					

Abbreviations: BAME, Black, Asian and Minority Ethnic; BTEC, Business and Technology Education Council; CI, confidence interval; GCSE, General Certificate of Secondary Education; ONC, Ordinary National Certificate; OR, odds ratio.

a Typically corresponding to formal education up to age 18.

b Typically corresponding to formal education up to age 16.

#### 2.3.3 Cancer fear

Cancer fear was assessed using 2 items relating to intensity (“How anxious do you feel when you think about cancer?” scored as “not at all,” “slightly,” “quite,” and “extremely”) and frequency (“In general, how often do you worry about getting cancer yourself?” scored as “never,” “occasionally,” “sometimes,” “often,” and “very often”), adapted from a previous survey.^23^ Three levels of cancer fear were created as in a previous study^12^^:^ “no cancer fear” (“not at all” anxious and “never” worried about cancer), “moderate cancer fear” (“slightly” anxious, or “occasionally” or “sometimes” worried), and “high cancer fear” (“quite” or “extremely anxious,” or “often” or “very often” worried; see Data S1).

#### 2.3.4 Cancer information avoidance

Cancer information avoidance was assessed using 3 “yes”/”no” items (adapted from^24^) asking about avoidance of 3 cancer information sources (stories in newspapers, magazines, or online; other people; TV programmes; see Data S2). Overall cancer avoidance was dichotomised into “no avoidance” (“no” on all 3 items) or “avoids at least one information source” (“yes” to at least one item).

### 2.4 Analyses

#### 2.4.1 Qualitative analysis

Open‐ended responses were transcribed and analysed using content analysis. JW, CV, and EA inductively coded the data, with any ambiguity resolved by discussion. When the initial codes were examined, they broadly fitted with the 5 dimensions of Leventhal's common sense model of illness representations (CSM),^25^ so this theory was used to refine the coding frame.

The CSM states that the illness representations of a health threat determine an individual's coping response to the threat.^25^ Illness representations have 5 attributes: “identity” refers to ideas about the (somatic) representation of an illness; “timeline” refers to beliefs about the timeframe of that illness and development over time; “causes” refers to beliefs about an illness' causes; “controllability” refers to the perceived controllability of the illness, and “consequences’ refers to representations of the anticipated consequences of the illness. According to the CSM, these attributes together determine the cognitive appraisal of a health threat, which—along with the emotional appraisal of the threat—determine an individual's behavioural response.^25^

We added 4 categories (“death (incurability),” a specific anticipated outcome of cancer that also has aspects of its expected development over time, “emotional response,” “social networks,” and “miscellaneous”), while “timeline” was removed because it was not deemed applicable to those without a diagnosis. Category definitions used here may therefore slightly differ from the definitions of the CSM (see Data S3). Following this refinement of the coding frame, EA coded the responses into themes and subthemes, with 10% of the coding checked by CV. Ambiguous responses (ie, those that could be interpreted in different ways) were classified as “miscellaneous.” “Don't know,” “nothing,” illegible, and refused responses were coded as missing. Responses covering more than one category were coded across all applicable categories. For example, “my wife died of breast cancer” was coded under “death” and “someone died of cancer.” RD second‐coded all responses, with any disagreements resolved through discussion with CV. Cohen's kappa for interrater reliability was .83, representing high agreement.^26^, ^27^

#### 2.4.2 Quantitative analysis

We described the prevalence of older adults' first associations of cancer using absolute numbers and percentages. We then conducted unadjusted and adjusted binary logistic regression analyses to investigate the sociodemographic distribution of holding the most common first association with cancer (“incurability or death”), and to examine the association of “death associations” with cancer fear and cancer information avoidance. We focused on the association of cancer with death because it formed the most homogenous theme in our inductive analysis and previous research has shown that fatalistic beliefs negatively affect cancer control strategies such as screening and early detection (see Section 1). Quantitative analyses were performed using IBM SPSS version 22.0 and an alpha level of *P* < .05.

## 3 RESULTS

### 3.1 Sample characteristics

After excluding those with a cancer diagnosis (n = 127), missing sociodemographic characteristics (n = 73) or missing data on the first association of cancer question (n = 159), 1105 participants were included for analysis. Mean age was 60 years (SD = 6.27) and 51% were male (Table 1). The majority were married (62%) and of White ethnic origin (94%), comparable to the general population in this age group in England (65% and 92%, respectively).^28^ About a quarter (26%) had no formal qualifications, compared with 25% in this age group in the general population.^28^ The regional distribution was similar to the general population.^29^ Most respondents (72%) had experience of cancer in close friends or family members. There were no significant differences in sociodemographic characteristics between those who were included in the analyses and those excluded due to missing data (results not shown).

### 3.2 First association with cancer

The results of the qualitative analysis of participants' first association with the word “cancer” are presented in Table 2. Overall, 86% of responses were coded under one theme only, while 14% were coded under multiple applicable themes. “Death” was the most frequent association with cancer (mentioned by 26%), followed by references to the controllability of cancer (25%), its identity (23%), emotional responses to cancer (15%), social networks (10%), and cancer consequences (8%). A small minority (4%) mentioned potential causes of cancer, such as smoking or genetics.

**Table 2:** Themes and subthemes of first associations with “cancer” (n = 1105)

Themes/Subthemes	n (%)^a^
Incurability/death	283 (25.6)
Identity	254 (23.0)
Negative beliefs about cancer	110 (9.9)
Cancer as a health condition	44 (4.0)
Type of cancer	39 (3.5)
Avoidance	18 (1.6)
Not bothered by cancer	33 (3.0)
Not specified	10 (0.9)
Emotional response	164 (14.8)
Negative	133 (12.0)
Positive/hopeful	16 (1.4)
Empathy	15 (1.4)
Causes	39 (3.5)
Smoking	16 (1.4)
Genetics	8 (0.7)
Others	15 (1.4)
Controllability	280 (25.3)
Survival	66 (6.0)
References to general treatment	119 (10.8)
References to specific treatments	44 (4.0)
Prevention and early detection	43 (3.9)
Cancer research or campaigns	8 (0.7)
Consequences of cancer	87 (7.9)
Physical	65 (5.9)
Social	10 (0.9)
Other types of consequences	20 (1.8)
Social networks	111 (10.0)
Someone they know (no elaboration)	56 (5.1)
Someone had a history of cancer	14 (1.3)
Someone died of cancer	41 (3.7)
Miscellaneous	36 (3.3)

a Total number of participants may not add up due to some responses being coded under multiple themes or subthemes.

In terms of cancer controllability, most respondents made references to cancer treatments (eg, “chemotherapy” or “surgery”), with fewer respondents referring to cancer survival or early detection. In terms of identity, most respondents had negative beliefs about cancer (eg, “horrible disease”), while fewer people had more neutral associations of cancer as “just another health condition.” Only very few people spontaneously reported cancer as something to be avoided (2%). Negative emotional responses (eg, “panic”) were much more common than positive, hopeful, or empathetic ones (eg, “hope people do survive”). About a third of those who referred to their social networks mentioned someone who had died from cancer. Finally, a small proportion associated cancer with its physical (eg, “pain”) or other consequences.

### 3.3 Sociodemographic distribution of having “death” as first association

We examined the sociodemographic distribution of having “death” as first association of cancer (Table 1). Those with no qualifications (38%; OR = 2.73; 95% CI, 1.78‐4.19) or lower levels of formal education (29%; OR = 1.85; 95% CI, 1.19‐2.88) were more likely to have “death” as a first association compared with those with a Bachelor's degree or higher (19%, reference). As predicted, there were no differences between respondents from the East (reference) and South of England, but respondents from the North (OR = 2.15; 95% CI, 1.31‐3.51) or Midlands (OR = 1.74; 95% CI, 1.03‐2.95), where cancer mortality rates are higher,^20^ were more likely to associate cancer with “death.” Those without experience of cancer in others were less likely to have death associations (23% vs 32%; OR = 0.69; 95% CI, 0.50‐0.94). There were no associations with age, gender, marital status, or ethnicity. These patterns remained the same in the adjusted analyses.

### 3.4 Cancer fear, cancer information avoidance, and death associations

Nearly half of participants were “not at all” anxious about cancer (43%) or “never” worried about it (49%; Data S2). When these measures were combined, about a third (36%) had no cancer fear, nearly half had moderate fear (46%), and 16% had high cancer fear. High cancer fear was associated with a higher likelihood of having “death” as a first association of cancer compared with no cancer fear in both unadjusted and adjusted analyses (41% vs 23%; OR(unadj) = 2.16; 95% CI, 1.47‐3.18; Table 1). Almost a quarter (23%) reported avoiding at least one source of cancer information, but there was no association between “death” associations and cancer information avoidance.

## 4 CONCLUSIONS

This was the first population‐based study to qualitatively examine first associations with cancer in a large sample of middle‐aged and older adults, for whom the threat of cancer is relevant due to their age. Despite improvements in cancer outcomes over recent decades, cancer was most commonly associated with death. Positive and hopeful associations, and references to modifiable risk factors, early detection, and survival, were infrequent.

This study extends and quantifies previous findings by Robb et al^15^ that initial responses to cancer tend to be negative, by indicating how common “death” and other negative responses to cancer are and by examining the sociodemographic patterning of these responses. Our study replicates the finding that almost a quarter of middle‐aged and older adults in England avoid cancer information.^12^ The proportion of participants who spontaneously reported death associations was also similar to the proportion agreeing to prompted statements that “cancer is a death sentence” found in previous quantitative studies in the UK.^3^, ^30^

Death associations were more common among those with lower levels of education, from regions with higher cancer mortality, and among those with previous experience of cancer in others. This is consistent with suggestions by other authors that fatalistic cancer attitudes may be the indirect consequence of under‐education and poverty, leading to a focus on day‐to‐day survival rather than health‐promoting behaviours, and potentially causing delays in help‐seeking for symptoms and poorer cancer outcomes.^31^, ^32^, ^33^, ^34^, ^35^ Fatalistic beliefs in families and communities may be reinforced by witnessing this cycle of poorer cancer outcomes.^31^, ^32^, ^36^ The “availability heuristic,” or the ease with which instances of cancer deaths can be brought to mind,^37^ may further reinforce fatalistic beliefs.

### 4.1 Clinical implications

Our study furthers our understanding of public attitudes to cancer. Leventhal's CSM states that the cognitive representation of a health threat (eg, “cancer means death”) is processed alongside the emotional representation of the health threat (eg, “fear”), and that these parallel processes jointly determine the behavioural response to the threat.^25^ Although previous studies suggest that people may be “in two minds” about cancer,^3^, ^15^ our study shows that the first response to cancer is often negative. Cancer fear and fatalism undermine cancer prevention and early detection behaviours,^7^, ^8^, ^9^, ^10^, ^11^, ^12^ and further research is needed to explore how these attitudes are formed and how they may be changed to increase the effectiveness of cancer control strategies, especially in groups with lower levels of education. Examples of effective interventions to address fatalistic attitudes in the US included video interventions aimed at targeting barriers to cancer screening or presenting narratives of cancer survivors.^38^, ^39^ These formats may be particularly useful for those from lower socio‐economic or ethnic minority backgrounds.^38^, ^39^

Besides negative beliefs, quantitative surveys have also shown high endorsement of positive beliefs about cancer. This could indicate that public beliefs about cancer are in transition; middle‐aged and older adults may be aware of the improvements in cancer survival that have happened over their lifetime, but this awareness may not yet have become part of their instinctive responses. Cancer attitudes may thus be shifting away from fatalistic beliefs and towards a view of cancer as a “chronic” condition.^40^

### 4.2 Study limitations

This study had some limitations. First, generalisability may be affected by the sample not being a random probability sample, although quota sampling was used to ensure similar study sample characteristics to those of the general population. Furthermore, it is unclear whether the question about first associations with cancer assessed more intuitive or more considered responses. Most participants gave very short, or even single‐word, answers, which may reflect the context of the structured interview in which the question was asked; this was different to the in‐depth interviews of the study by Robb et al.^15^ Additionally, responses were recorded by the interviewer, and social desirability may have affected participants' answers. Future research could explore whether responses are similar when recorded by the participants themselves. Due to the cross‐sectional nature of the study, we cannot draw any conclusions about the temporal or causal nature of the relationships; longitudinal studies would need to examine how people's first associations may evolve over time to reflect improvements in cancer care and curability. Finally, previous research has shown that attitudes may differ between cancer types^40^; future research could ask about specific types of cancer.

In conclusion, a significant proportion of middle‐aged and older adults in England associate “cancer” with “death,” especially those from lower socio‐economic backgrounds or regions with higher cancer mortality. These fatalistic associations may undermine engagement with cancer prevention and early detection initiatives. Efforts are needed to understand how associations with “cancer” are formed, and how unduly negative associations may be altered to improve cancer outcomes.

## CONFLICT OF INTEREST

The authors declare that they have no competing interests.

## CONTRIBUTORS

CV and JW conceived of the study and its design and were responsible for data collection. EA, JW, and CV developed the coding framework, which was refined with input from RD. EA, RD, and CV conducted the qualitative coding. EA and RD conducted the quantitative analyses, with input from CV. EA and RD prepared the final manuscript, with input from JW and CV. All authors have seen and approved the final manuscript before submission.

## Supporting information

Data S1. Supporting Information
