# Supplementary material for: Understanding middle‐aged and older adults' first associations with the word “cancer”: A mixed methods study in England
Source: Psychooncology. 2017 Nov 7;27(1):309–15. doi: 10.1002/pon.4569 (PMC5813269; doi:10.1002/pon.4569)
Supplement: Supplementary file 1 — Data S1. Supporting Information [file PON-27-309-s001.zip › Online supplement 1.docx]

**Online supplement 1 to “Understanding older adults’ first associations with the word ‘cancer’: a mixed methods study in England”**

By Edelyn Agustina, Rachael Dodd, Jo Waller, and Charlotte Vrinten.

**Categorisation of the cancer fear items**

|  | Cancer worry | | | | | |
| --- | --- | --- | --- | --- | --- | --- |
| Cancer anxiety | Never | Occasionally | Sometimes | | Often | Very often |
| Not at all | No fear |  |  | |  |  |
| Slightly |  | Moderate fear | | |  |  |
| Quite a bit |  |  | |  | High fear | |
| Extremely |  |  | |  |  |  |

Reproduced with permission from Vrinten C, Boniface D, Lo SH, et al. (2017). Does psychosocial stress exacerbate avoidant responses to cancer information in those who are afraid of cancer? A population-based survey among older adults in England. Psychology & Health, Epub April 2017. DOI: 10.1080/08870446.2017.1314475. <http://dx.doi.org/10.1080/08870446.2017.1314475>.
