# Supplementary material for: Understanding middle‐aged and older adults' first associations with the word “cancer”: A mixed methods study in England
Source: Psychooncology. 2017 Nov 7;27(1):309–15. doi: 10.1002/pon.4569 (PMC5813269; doi:10.1002/pon.4569)
Supplement: Supplementary file 1 — Data S1. Supporting Information [file PON-27-309-s001.zip › Online supplement 2.docx]

**Online supplement 2 to “Understanding older adults’ first associations with the word ‘cancer’: a mixed methods study in England”**

By Edelyn Agustina, Rachael Dodd, Jo Waller, and Charlotte Vrinten.

Descriptive statistics for participants’ attitude towards cancer (n=1105)

|  | **n (%)** |
| --- | --- |
| **Cancer intensity: How anxious do you feel when you think about cancer?** | |
| Not at all  Slightly anxious  Quite anxious  Extremely anxious  Don’t know/refused | 472 (42.7)  445 (40.3)  135 (12.2)  35 (3.2)  18 (1.6) |
| **Cancer frequency: In general, how often do you worry about getting cancer yourself?** | |
| Never  Occasionally  Sometimes  Often  Very often  Don’t know/refused | 540 (48.9)  385 (34.8)  125 (11.3)  29 (2.6)  14 (1.3)  12 (1.1) |
| **Cancer fear: Cancer anxiety and cancer worry combined** | |
| No cancer fear  Moderate cancer fear  High cancer fear  Don’t know/refused | 393 (35.6)  512 (46.3)  173 (15.7)  27 (2.4) |
| **Cancer avoidance 1: Do you avoid reading stories about cancer in newspapers, a magazine or online?** | |
| Yes  No  Don’t know/refused | 187 (16.9)  861 (77.9)  57 (5.2) |
| **Cancer avoidance 2: Do you avoid talking to other people about cancer?** | |
| Yes  No  Don’t know/refused | 128 (11.6)  969 (87.7)  8 (0.7) |
| **Cancer avoidance 3: Do you avoid watching programmes about cancer on TV?** | |
| Yes  No  Don’t know/refused | 229 (20.7)  821 (74.3)  55 (5.0) |
| **Overall cancer avoidance** |  |
| No avoidance  Avoids one of the three behaviours  Avoids two of the three behaviours  Avoids all behaviours  Don’t know/refused | 755 (68.3)  86 (7.8)  92 (8.3)  78 (7.1)  94 (8.5) |
