# Supplementary material for: Understanding middle‐aged and older adults' first associations with the word “cancer”: A mixed methods study in England
Source: Psychooncology. 2017 Nov 7;27(1):309–15. doi: 10.1002/pon.4569 (PMC5813269; doi:10.1002/pon.4569)
Supplement: Supplementary file 1 — Data S1. Supporting Information [file PON-27-309-s001.zip › Online supplement 3.docx]

**Online supplement 3 to “Understanding older adults’ first associations with the word ‘cancer’: a mixed methods study in England”**

By Edelyn Agustina, Rachael Dodd, Jo Waller, and Charlotte Vrinten.

Description and examples of themes and subthemes for content analysis of participants’ first associations with cancer

| **Themes/Subthemes** | **Description** | **Examples** |
| --- | --- | --- |
| **Incurability (Death)** | Statements related to death or states/implies that cancer is incurable | ‘Death’, ‘No cure’, ‘My mum died of cancer’ |
| **Identity** | Statements related to beliefs about cancer (Does not include beliefs about cancer treatment—see ‘references to general treatment’) | |
| Negative beliefs about cancer | Describes cancer as something negative or serious or with negative word(s) | ‘Bad’, ‘Horrible disease’, ‘Scary’ |
| Cancer as a health condition | Describes cancer as a state of health or the symptoms, progression or appearance of cancer without positive or negative evaluation | ‘Illness’, ‘Lumps’, ‘Deformed cells’ |
| Type of cancer | States a specific type of cancer or asks what type of cancer it is | ‘Breast cancer’, ‘What sort’, ‘Where is it’ |
| Avoidance | Describes cancer as something to be avoided or does not want to get cancer | ‘Try not to think’, ‘I hope I don’t get it’ |
| Not bothered by cancer | Perceives cancer as something not given a thought about (but not actively avoiding it) | ‘Don’t think about it’, ‘Not thought about it’, ‘Take it as it comes’ |
| Not specified | Perceives cancer as something that does not fall into any of the above subthemes | ‘Big C’, ‘Very common’ |
| **Emotional response** | Statements related to how individual feel or would feel about cancer | |
| Negative | Expresses negative emotional reactions or feelings towards cancer | ‘Fear’, ‘Panic’, ‘Why me’ |
| Positive/Hopeful | Provides encouraging or positive comments or are not negatively affected by cancer | ‘Remain positive’, ‘Fight it’, ‘Not scared’ |
| Empathy | Expresses sympathy to affected individuals | ‘Sympathy’, ‘Hope people do survive’ |
| **Causes** | Statements related to what individuals think causes/caused cancer | |
| Smoking | Mentions smoking or relates smoking as a cause of cancer | ‘Smoking’, ‘Increased if not given up smoking’ |
| Genetics | Attributes cancer to individual’s genetics or perceives cancer as something heritable | ‘Family history’, ‘Genetics’ |
| Others | Mentions other causes of cancer besides smoking and genetics | ‘Fate’, ‘Getting old’, |
| **Controllability** | Statements related to how cancer is/should be dealt with |  |
| Survival | Indicates that cancer is curable or that there is chance of surviving or questions curability | ‘Curable’, ‘Not death anymore’, ‘Will I survive’ |
| References to general treatment | Mentions treatment related terms, description of treatment or questions whether cancer is treatable. Note: Treatment/treatability and cure/curability are not the same | ‘Hospital’, ‘Horrible treatment’, ‘How to treat’, ‘seek a doctor’ |
| References to specific treatments | Mentions specific types of cancer treatment | ‘Chemotherapy’, ‘Radiation treatment’, ‘Surgery’ |
| Prevention and early detection | Mentions prevention or views early detection or treatment of cancer to be important | ‘Preventing it’, ‘Early diagnosis’, ‘Seek treatment ASAP’ |
| Cancer research or campaigns | Mentions cancer research, cancer related organisations or campaigns to cure or raise awareness about cancer | ‘Cancer research’, ‘Macmillan’, ‘Pink ladies running around the streets’ |
| **Consequences of cancer** | Statements related to perceived consequences of having cancer, with the exception of responses that mentions death—see ‘death’ | |
| Physical | Mentions negative changes to bodily parts as a result of cancer or state of poor health or pain | ‘Pain’, ‘being ill’, ‘losing your hair’ |
| Social | Expresses concerns towards close ones if they were to have cancer | ‘Family you leave behind’, ‘Disruption to family life’ |
| Other types of consequences | Mentions other types of consequences of cancer that does not fall into any of the above subthemes | ‘Suffering’, ‘Life changing’, ‘Tough time ahead’ |
| **Social networks** | Statements that mentioned family members, friends or people he/she knows | |
| Someone they know | Mentions family members or friends or people he/she knows without any elaboration | ‘Family’, ‘My dad’, ‘People I know’ |
| Someone had a history of cancer | Mentions family members or friends who either has or has had cancer | ‘Brother had it’, ‘Friends who have had cancer’, ‘People who have it’ |
| Someone died of cancer | Mentions family members or friends who died of cancer (All responses will also be coded under theme ‘incurability (death)’) | ‘Wife death’, ‘Lost ones’, ‘Losing family members’ |
| **Miscellaneous** | Statements that are ambiguous or does not match any of the above themes | ‘Work’, ‘Bad design’, ‘Support’ |
| **Missing** | Participants responded don’t know, nothing, refused to answer or if response is illegible for transcription | |
| Don’t know/Nothing | Replies don’t know, nothing or not applicable | ‘Don’t know’, ‘No’, ‘Nothing’, ‘NA’ |
| Illegible | Unable to read response due to bad handwriting |  |
| Refused | Does not answer question at all |  |
